# Enzymatically enhanced ultrastructure expansion microscopy unlocks expansion of *in vitro Toxoplasma gondii* cysts

**DOI:** 10.1128/msphere.00322-24

**Published:** 2024-08-27

**Authors:** Kseniia Bondarenko, Floriane Limoge, Kayvon Pedram, Mathieu Gissot, Joanna C. Young

**Affiliations:** 1 Institute of Immunology and Infection Research, School of Biological Sciences, Ashworth laboratories, University of Edinburgh, Edinburgh, United Kingdom; 2 U1019-UMR 9017-CIIL-Center for Infection and Immunity of Lille, University of Lille, CNRS, Inserm, CHU Lille, Institut Pasteur de Lille, Lille, France; 3 Janelia Research Campus, Howard Hughes Medical Institute, Ashburn, Virginia, USA; Indiana University School of Medicine, Indianapolis, Indiana, USA

**Keywords:** expansion microscopy, apicomplexan parasites, host–parasite relationship, chronic infection, Toxoplasma

## Abstract

Expansion microscopy (ExM) is an innovative approach to achieve super-resolution images without using super-resolution microscopes, based on the physical expansion of the sample. The advent of ExM has unlocked the detail of super-resolution images for a broader scientific circle, lowering the cost and entry skill requirements for the field. One of its branches, ultrastructure expansion microscopy (U-ExM), has become popular among research groups studying apicomplexan parasites, including the acute stage of *Toxoplasma gondii* infection. Here, we show that the chronic cyst-forming stage of *Toxoplasma*, however, resists U-ExM expansion, impeding precise protein localization. We then solve the *in vitro* cyst’s resistance to denaturation required for successful U-ExM. As the cyst’s main structural protein CST1 contains a mucin domain, we added an enzymatic digestion step using the pan-mucinase StcE prior to the expansion protocol. This allowed full expansion of the cysts in fibroblasts and primary neuronal cell culture without disrupting immunofluorescence analysis of parasite proteins. Using StcE-enhanced U-ExM, we clarified the localization of the GRA2 protein, which is important for establishing a normal cyst, observing GRA2 granules spanning across the CST1 cyst wall. The StcE–U-ExM protocol allows accurate pinpointing of proteins in the bradyzoite cyst, which will greatly facilitate investigation of the underlying biology of cyst formation and its vulnerabilities.

**IMPORTANCE:**

*Toxoplasma gondii* is an intracellular parasite capable of establishing long-term chronic infection in nearly all warm-blooded animals. During the chronic stage, parasites encapsulate to form cysts predominantly in neurons and skeletal muscle. Current anti-*Toxoplasma* drugs do not eradicate chronic parasites, leaving a reservoir of infection. The cyst is critical for disease transmission and pathology, yet it is harder to study, with the function of many chronic-stage proteins still unknown. Ultrastructure expansion microscopy, a new method to overcome the light microscopy’s diffraction limit by physically expanding the sample, allowed in-depth studies of acute *Toxoplasma* infection. We show that *Toxoplasma* cysts resist expansion using standard protocol, but an additional enzymatic digestion with the mucinase StcE allows full expansion. This protocol offers new avenues for examining the chronic stage, including precise spatial organization of cyst-specific proteins, linking these locations to morphological structures, and detailed investigations of components of the durable cyst wall.

## INTRODUCTION

Expansion microscopy (ExM) is a sample preparation protocol that allows imaging to bypass the diffraction limit of conventional light microscopes (~200 nm) by physically expanding the biological sample embedded in a gel (1). As an affordable and accessible technique when compared to expensive and laborious electron microscopy (EM), ExM has been transformative across diverse research fields, allowing nanoscale resolution of protein localization using light microscopes.

Among the numerous ExM protocols developed so far, ultrastructure expansion microscopy (U-ExM) (2) gained popularity in the field of parasitology due to the improved preservation of subcellular structures (3). U-ExM allows 4.0–4.5× expansion (one-dimensional expansion factor, termed the expansion factor going forward) of the biological specimen (~90-fold increase in volume) in one step with the preservation of the proteins, in contrast to other approaches which leave only the protein footprint after proteolytic digestion (2, 4). U-ExM therefore enables all-protein staining using non-specific protein dyes like N-hydroxysuccinimde (NHS) esters (5). U-ExM has been widely adopted in apicomplexan research, generating new insights in to diverse parasite life cycle stages and clarifying the localization of over 80 proteins in *Toxoplasma gondii* (6–14), *Cryptosporidium parvum* (15, 16), and *Plasmodium* spp. (5, 17–26). However, investigations of the chronic *Toxoplasma gondii* cyst have lagged behind.

*Toxoplasma* is a single-celled intracellular parasite capable of establishing long-term chronic infection in nearly all warm-blooded animals, including humans (27). *Toxoplasma* has a complex life cycle and differentiates between two non-sexual forms within intermediate hosts (28). During the acute stage of infection, the parasites replicate rapidly as so-called tachyzoites and disseminate around the host’s body. To establish long-term infection, parasites differentiate to slow-growing bradyzoites and develop cysts predominantly in the central nervous system and skeletal muscle. Reactivation of latent cysts can cause fatal encephalitis in the immunocompromised or recurrent ocular disease (retinochoroiditis) (29).

*Toxoplasma* tissue cysts are intracellular and are spherical structures 5–100 μm in diameter (28), containing a collection of tightly packed bradyzoites. The cyst wall is between 250 and 850 nm thick and composed of a granular layer of proteins and carbohydrates underneath the limiting membrane [a modified version of the parasitophorous vacuole (PV) membrane, which forms as the parasite invades the host cell] (28). The accumulation of glycoproteins in the cyst wall ensures structural and chemical rigidity (30) and is thought to allow parasite transmission to infect a new host following ingestion of undercooked meat.

*Toxoplasma* cysts can also be generated *in vitro* in tissue culture, which has been proven to be a valuable model for studying cyst biology. *Toxoplasma* tachyzoites differentiate to bradyzoites under stress conditions and exposure alkaline stress is the most commonly used method (31, 32). However, recent advances have improved the model with spontaneous differentiation to bradyzoites in myotubes and neurons, producing longer-lasting cysts (14–28 days) (33, 34).

The cyst wall contains markers exclusively expressed during the chronic stage (35). One of the early chronic-stage markers, CST1, is the major structural element of the cyst wall, and parasites lacking *cst1* form fragile cysts (30). CST1 has a highly O-glycosylated mucin domain which binds to the lectin Dolichos biflorus agglutinin (DBA) (30, 36). The structure of CST1’s mucin domain includes 20 threonine-rich tandem repeats which undergo O-GalNAc glycosylation. This glycosylation process is initiated by a group of enzymes known as polypeptide N-acetylgalactosaminyltransferases (30, 36). Previous studies using electron microscopy have shown that CST1 is expressed in the granular material in the cyst wall under the limiting membrane (37). It has been hypothesized that the high glycosylation of the CST1 mucin domain could act as a bonding agent for other proteins associated with the cyst wall, such as dense granule proteins (GRAs) (38).

*Toxoplasma* GRAs are secreted from dense granules into the PV lumen or out into the host cell and perform a variety of functions during infection, including mediating host–parasite interactions, modification of the PV, and establishment of an intravacuolar network (IVN) of highly curved nanotubules (39). The latter is involved in connecting tachyzoites in the PV and in the salvage of lipids (40, 41) and cytosolic proteins (42) from the host. Some of the GRA proteins, especially those associated with the IVN network (GRA2, GRA6, GRA4, and GRA12), relocalize to the forming cyst wall and impact CST1 localization (38). Of these, GRA2 is essential for IVN formation (43), acute virulence, and cyst formation (44, 45), yet details of its function during the chronic stage remain unclear. GRA2 appears to have a dynamic cyst-wall localization being present in early cysts at day 7 (D7) and D10, with its location overlapping with the CST1 signal (38), while being absent from the wall in late cysts (46).

Here, we apply U-ExM to *Toxoplasma* tissue cysts produced *in vitro*, demonstrating that the original protocols do not completely expand these structures. This problem is solved by the addition of an enzymatic digestion step with mucin-selective protease StcE (47) during the sample preparation. StcE cuts through the mucin domains of the major structural protein of the cyst wall CST1, allowing the cyst to expand fully while preserving the CST1 signal in immunofluorescence assays. We used this method to assess the co-localization of CST1 and GRA2 in the cyst wall and observed distinct localization of GRA2 at this higher resolution. The addition of the StcE step to the U-ExM protocol opens new avenues to precisely localize proteins in bradyzoites, as exemplified here with the proteins associated with the cyst wall, aiding in understanding their role in cyst formation and growth.

## MATERIALS AND METHODS

### Cell culture

Primary human foreskin fibroblasts (HFFs) (American Type Culture Collection) were maintained in Dulbecco’s modified Eagle’s medium (DMEM, Sigma-Aldrich) with 4.5-g/L glucose, 25-mm HEPES, and 1% vol/vol GlutaMAX (Gibco) supplemented with 10% vol/vol fetal bovine serum (FBS, Gibco) at 37°C with 5% CO_2_.

### Parasite strains and culture

*Toxoplasma* gondii ME49Δ*ku80*Δ*hxgprt* (48) and 76K tachyzoites were propagated *in vitro* in HFFs using DMEM supplemented with 2% v/v heat-inactivated FBS, 1% v/v glutaMAX, and 1% v/v penicillin-streptomycin solution (Gibco). Tachyzoites were grown in ventilated tissue culture flasks at 37°C and 5% CO_2_. Prior to infection, intracellular parasites were isolated by syringe passage with 23-gauge blunt needles (SAI Infusion Technologies) and filtration through a 5-µm membrane filter (Sartorius).

### Parasite infections

For confocal and U-ExM imaging, HFFs were seeded on 13-mm no. 1.5 coverslips (SLS) in 24-well plates and grown to confluent monolayers for at least 7 days old before infection. For tachyzoite samples, cells were infected at a multiplicity of infection (MOI) of 1 for 24 h. For bradyzoite cultures, HFF monolayers were infected with tachyzoites at an MOI of 1 for 3.5 h, followed by media change to filtered RPMI 1640 media (Sigma-Aldrich) supplemented with 1% vol/vol FBS and 50-mM HEPES (Sigma-Aldrich) and brought to pH 8.2. The bradyzoite culture was grown with ambient CO_2_ at 37°C for 7 days with daily media changes. All samples were fixed in 4% formaldehyde (FA, Thermo Scientific) for 15 min at room temperature (RT), followed by 3 × 1 min phosphate-buffered saline (PBS) wash.

*T. gondii* infection of primary neuronal culture obtained from the hippocampus of postnatal rats was performed as described previously (33). Briefly, after the dissection of the brains, hippocampi were mechanically dissociated and resuspended in Neurobasal A, a medium supplemented with GlutaMAX and B27 neural supplement with antioxidants (Gibco). Cells were plated at a density of 100,000 cells/cm^2^ in poly-L-lysine coated 24-well plates containing coverslips. Brain cells were grown for 14 days before infection. Each well was infected by 1 × 10^4^ tachyzoites of the 76K strain resuspended in the Neurobasal A medium. The culture was grown for an extra 14 days to obtain fully mature cysts as described in reference (33).

### Standard immunofluorescence assays

For immunofluorescence assays, fixed cells were permeabilized with 0.1% Triton X-100 (Sigma-Aldrich) in PBS for 2 min at RT for tachyzoites or 0.2% Triton X-100, 0.1% glycine (Sigma-Aldrich), and 0.2% bovine serum albumin (BSA) in PBS for 20 min on ice for bradyzoites. Samples were then incubated in blocking solution (2% BSA in PBS) for 1 h, followed by 45-min incubation with antibodies/stains (diluted in blocking solution).

Infected cells, depending on the experiment, were stained using antibodies and dyes listed in Table 1. coverslips were washed 3× with PBS after primary and secondary antibody/stain incubations, and coverslips were subsequently washed and mounted with Vectashield Antifade softset mountant (H-1000, Vector Laboratories). Parasites were visualized on a Zeiss LSM980 laser confocal microscope.

**TABLE 1:** Key resources table^*a*^

Category	Reagents	Catalog no. (LOT)	Manufacturer	Additional information
U-ExM protocol reagents	AA, 40%	A4058	Sigma-Aldrich	
	APS	A3678-25G	Sigma-Aldrich	
	FA, 37%	F8775	Sigma-Aldrich	
	N,N′-methylenbisacrylamide, 2% (Bis)	M1533	Sigma-Aldrich	
	Sodium chloride (NaCl)	S/3160/63 (2053700)	Thermo Fisher	
	SDS, 20%	AM9820	Thermo Fisher	
	SA	R624	AKSci	
	TEMED	T7024	Sigma-Aldrich	
	Tris base	BP152-1	Thermo Fisher	
	Tween 20	P1379	Sigma-Aldrich	
	Poly-D-lysine	A38904-01 (959890E)	Thermo Scientific	
	StcE mucinase, 20 µM (MW 98 kDa)	Gift from Kayvon Pedram (49)		Commercially available from Merk, Cat N SAE0202
Dyes, stains, antibodies, and mountants	DAPI (dilactate)	D3571	Invitrogen	1:1,000 (std), 1:100 (ExM)
	DBA, biotinylated	B-1035 (ZH1209)	Vector Laboratories	1:2,500 (std), 1:1,000 (ExM)
	Anti-GRA2 mouse	TG17-179 GRA2 #BIO.018.5 (vt210324a-4939)	Biotem	1:1,000 (std), 1:300 (ExM)
	Anti-SAG1 rabbit	ab138698 (GR3426284-1)	Abcam	1:1,000 (std)
	Anti-CST1 rabbit	Gift from Louis Weiss (30, 37)	–^*b*^	1:200 (std), 1:100 (ExM)
	Anti-TgGAP45 rabbit	Gift from Dominique Soldati-Favre (50)	–	1:10,000 (std), 1:7,000 (ExM)
	Anti-MAG1 mouse	Gift from Louis Weiss (51)		1:250 (ExM)
	AlexaFluor 488 goat anti-mouse superclonal	A28175 (2126853)	Thermo Scientific	1:1,000 (std), 1:300 (ExM)
	AlexaFluor 568 goat anti-mouse	A-11004 (1862187)	Thermo Scientific	1:1,000 (std), 1:300 (ExM)
	AlexaFluor 488 goat anti-rabbit	A-21206 (1981155)	Thermo Scientific	1:1,000 (std), 1:300 (ExM)
	AlexaFluor 568 goat anti-rabbit	A-11036 (1924788)	Thermo Scientific	1:1,000 (std), 1:300 (ExM)
	Atto 647N goat anti-rabbit	40839–1ML-F (BCBV5056)	Sigma-Aldrich	1:1,000 (std), 1:300 (ExM)
	NHS ester, Atto 565 conjugate NHS ester, Atto 594 conjugate	72464 08741	BioReagent	1:1,500 (std), 1:3000 (ExM)
	Streptavidin, AlexaFluor 488 conjugate	S11223 (2390711)	Invitrogen	1:1,000 (std), 1:300 (ExM)
	Streptavidin, AlexaFluor 568 conjugate	S11226 (2397941)	Invitrogen	1:1,000 (std), 1:300 (ExM)
	Mounting media Vectashield Antifade	H-1000–10 (ZE0806)	Vector Laboratories	
Tissue culture media and reagents	Dulbecco’s modified Eagle’s medium	61965–059 (2629747)	Thermo Fisher	
	RPMI 1640 media	R6504 (SLCM4599)	Sigma-Aldrich	
	Fetal bovine serum	10500–064 (2574402H)	Thermo Fisher	
	Bovine serum albumin	A9637 (SLCL4070)	Sigma-Aldrich	
	HEPES	H4034 (SLCG6096)	Sigma-Aldrich	
	Penicillin-streptomycin solution	15140122	Thermo Fisher	
	GlutaMAX	35050–061 (2523105)	Thermo Fisher	
	Formaldehyde, 16%	28908	Thermo Fisher	
	Glycine	50046 (SLCG0879)	Sigma-Aldrich	
	Triton X-100	T8787 (SLCJ6163)	Sigma-Aldrich	
Tissue culture and staining consumables	Blunt needles 23G	847.356.0321	SAI Infusion Technologies	
	Cellulose acetate membrane filter, 5 µm	SLSV025LS	Sartorius	
	Coverslips no. 1.5, diameter 13 mm	MIC3336	SLS	
	Press-to-seal silicone isolator (13 mm × 0.8 mm)	GBL666507-25EA	Sigma-Aldrich	
	35-mm-high glass-bottom petri µ-dishes	81148	Ibidi	

^*a*^ AA, acrylamide; APS, ammonium persulfate; DAPI, 4′,6-diamidino-2-phenylindole; DBA, Dolichos biflorus agglutinin; FA, formaldehyde; MW, molecular weight; SA, sodium acrylate; std, standard immunofluorescence analysis; TEMED, Tetramethylethylenediamine.

^*b*^ ‘–’, no company.

### U-ExM

The first detailed U-ExM protocol was published in 2021 (52). For reagents list, see Table 1. Briefly, infected HFFs or primary neuronal cultures on coverslips were first fixed (4% FA, 15 min) and then permeabilized (tachyzoites: 0.1% Triton X-100 in PBS for 2 min at RT; bradyzoites: 0.2% Triton X-100, 0.1% glycine, and 0.2% BSA in PBS for 20 min on ice). The Triton permeabilization step was necessary for antibodies to penetrate parasitophorous vacuole/cyst wall and allow intraparasite antigen detection. Samples were then incubated in protein cross-linking prevention solution (final concentration 1.4% FA/2% acrylamide) at 37°C with no shaking overnight. To obtain fully expanded cysts, samples were incubated with 100-nM StcE mucinase in PBS [98 kDa, gift from Kayvon Pedram (49)] for 4 h at 37°C with no shaking before the protein cross-linking prevention step. StcE is also commercially available (SAE0202, Merck). Coverslips were briefly washed 3× with PBS and prepared for gelation.

The gelation solution consisted of a monomer solution, topped up with TEMED (Sigma-Aldrich) and APS (Sigma-Aldrich) final solutions just before the sample application. (Table 2). All components were stored at −20°C and kept on ice until just before use.

**TABLE 2:** Expansion microscopy buffers

Buffer	Reagent	Stock concentration	Final concentration
FA/AA mix in PBS	PFA	37%	1.4%
	AA	40%	2%
StcE mucinase enzyme	StcE	20 µM	0.1 µM
Denaturation buffer	SDS	350 mM	200 mM
	NaCl	5,000 mM	200 mM
	Tris	1,000 mM	50 mM
	
Monomer solution	SA	38%	19%
	AA	40%	10%
	BIS	2%	0.1%
	PBS	10×	1×
Gelling solution top-up 1	TEMED	100%	10%
Gelling solution top-up 2	APS	100%	10%

The gelation chamber was adapted from reference (53) and was prepared as follows (Fig. S1). A clean press-to-seal silicone isolator (13-mm diameter × 0.8-mm depth, GBL666507, Sigma-Aldrich) was mounted on parafilm-wrapped coverglasses (three coverglasses were stacked together before wrapping to make the structure more rigid) (Fig. S1A and B). The device was put on a wetted piece of white roll paper, stretched in a plastic chamber (e.g., a 24-well plate lid), placed for 5 min in −20°C freezer, and then kept on ice (Fig. S1C).

Fresh gelation solution (120 µL) was placed into the well created by a silicon isolator. Immediately afterward, the sample coverslip was dabbed from the excess PBS and the cells were placed down on the top of the well. The gelation chamber was left for 5 min on ice to aid monomer permeation and then placed into an incubator at 37°C for 1 h for polymerization.

Once polymerized, the silicone isolator was peeled off. The coverslips were lifted from the parafilm-covered coverglass using tweezers, put in a six-well plate (one gel/well), topped with 2-mL denaturation solution (Table 2), and left for 15 min on a rocker. (By this time, the gels should have detached; if not, they were left on the rocker for another 5–10 min.) Detached gels were placed in in a 1.5-mL Eppendorf tube with the fresh denaturation solution and were incubated for 1.5 h at 95°C in a heating block with no shaking. After denaturation, the gels were then expanded in beakers with 250- to 300-mL ddH_2_O overnight (first round).

On the next day, the gels underwent 2 × 15 min incubation in PBS (no shaking) to shrink in size and then were cut to the needed shape using scalpel blade (to fit a well in a 24-well plate). Note that the gels shrink further during the staining process. Gels were then blocked for 1 h in a blocking solution (2% BSA in PBS) at 37°C while shaking and then incubated with the primary and secondary antibodies or stains for 2.5 h in the same conditions, with 3 × 10 min washes in PBS–0.1% Tween 20 solution on a rocker at RT in between incubation steps (during the secondary antibody incubation, the plates were wrapped in foil to prevent photobleaching). During the second round of expansion, the samples were then expanded at 2 × 30 min in 250- to 300-mL ddH_2_O, 30 min, and then left in fresh ddH_2_O overnight in the area protected from light.

Infected cells, depending on the experiment, were stained using antibodies and dyes listed in Table 1. Parasites were visualized on a Nikon Ti2 CSU-W1 Spinning Disk microscope or a Zeiss LSM980 laser confocal microscope in 35-mm-high glass-bottom petri µ-dishes (Ibidi, 81158, can be reused), covered in-house with poly-D-lysine (Thermo Scientific) for 1 h, followed by 3× ddH2O washes, and then dried.

### Image acquisition

Imaging was performed in Centre Optical Instrumentation Laboratory, University of Edinburgh. Two microscopes were used to visualize expanded samples: Nikon Ti2 CSU-W1 Spinning Disk Confocal (objective: ×100: Plan Apo TIRF, Oil, 1.45 NA) and Zeiss LSM980 laser-scanning confocal microscope (objective: ×100: Alpha Plan Apochromat, Oil, 1.45 NA, DIC). Initially, Nikon Ti2 CSU-W1 Spinning Disk Confocal was used for faster gel imaging to avoid potential gel drift. However, using Ibidi glass-bottom dishes with lids and poly-D-lysine covering substantially reduced the drift when imaged on the more sensitive Zeiss LSM980 laser-scanning confocal microscope. Thus, all consequent imaging was performed using Zeiss (without Airyscan 2 mode).

In the absence of a ×100 water objective (which is most suitable as the refractive index of expansion gels closely matches that of water), we used 100× oil, which still yielded excellent performance as the cysts were located in a monolayer of cells in close proximity to the dish coverslip.

### Image processing and analysis

The images were exported using either Zen Blue software (Zeiss) or ImageJ. The brightness was adjusted for display purposes, but the measurements were taken on raw unprocessed files. Data analysis and statistical tests (*t*-tests) were performed in GraphPad Prism software.

The expansion factor was determined by the non-blinded manual measurement and subsequent comparison of the cross-section length of parasite nuclei stained with 4′,6-diamidino-2-phenylindole nuclear stain (along the longest axis) in the tachyzoites before (three monolayer coverslips) and after expansion (three gels) using ImageJ line tool. Some cysts required two to three projections to display full thickness of its nuclei (to define the longest diameter, full projection of a single nucleus is required). Dense packing of parasites inside the cyst caused some nuclei to overlap in image projections, preventing automation of nuclei diameter measurements. Overlaying nuclei were excluded from the analysis.

Relative localization of GRA2 and CST1 in images of StcE–U-ExM HFFs and neuronal samples (non-blinded) was performed in ImageJ by measuring the distribution of gray values in GRA2 and CST1 channels across the set distance [line across the cyst wall , thickness 1]. Extracted measurements were then normalized (min–max), each to the highest gray value within the channel, and then plotted against the measured distance using GraphPad Prism software.

## RESULTS

### Standard ultrastructure expansion microscopy protocol does not fully expand *T. gondii* cysts

Standard U-ExM protocols have been successfully applied to extracellular *Toxoplasma* and tachyzoites within infected cells [reviewed in reference (3)]. To assess whether this method could be applied to *Toxoplasma* cysts, we first compared U-ExM protocol on *Toxoplasma*-infected cells grown under standard conditions (tachyzoites) or under pH stress to induce conversion to bradyzoites.

**Fig 1:**
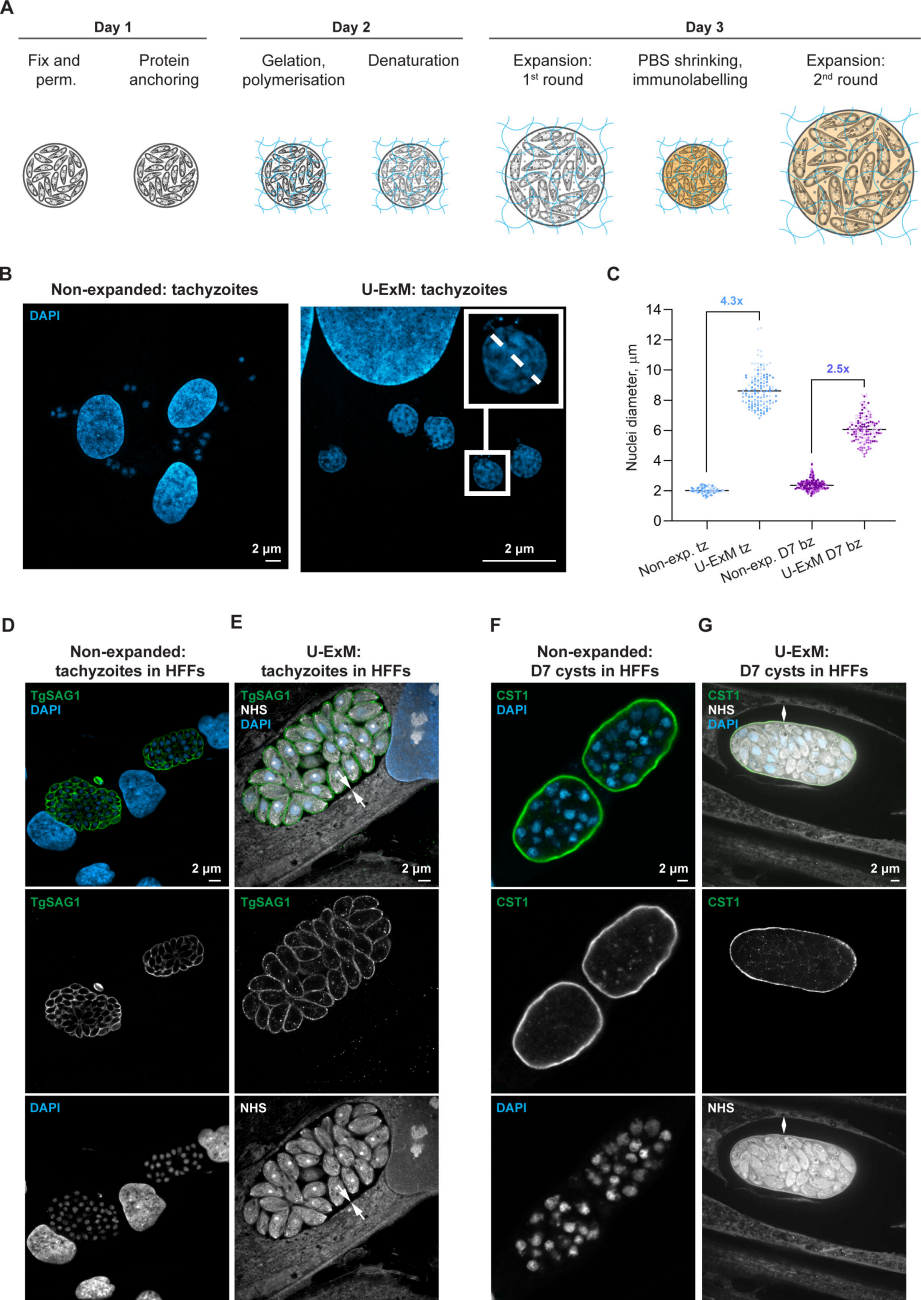
Standard U-ExM protocol expands intracellular *T. gondii* tachyzoites but fails to expand the cyst wall. (**A**) An overview of the standard U-ExM protocol workflow. (**B**) Confocal images of HFF monolayer infected with type II ME49*Δku80Δhxgprt* tachyzoites, fixed 24 h pi, and labeled with DAPI nuclear stain. Large nuclei belong to host cells; smaller nuclei are tachyzoite nuclei. Insert shows an example of a tachyzoite nuclei measurement along its longest diameter to calculate the one-dimensional expansion factor (termed simply expansion factor going forward) of the U-ExM sample. (**C**) The expansion factor was determined by comparison of the average cross-section of tachyzoite (blue) and bradyzoite (violet) nuclei stained with DAPI in non-expanded samples versus U-ExM samples. The graph displays three independent gels or coverslips (for non-expanded samples) per condition (dots depict single measurements, and each color shade indicates measurements from the same sample). The tachyzoite expansion factor is 4.3× but only 2.5× for bradyzoites (for both unpaired *t*-tests, *P* < 0.0001) (*n*_non-exp. tz_ = 79, *n*_exp. tz_ = 131, *n*_non-exp. bz_ = 201, *n*_exp. bz_ = 112). (**D and E**) Immunofluorescence images of HFFs infected with ME49*Δku80Δhxgprt* tachyzoites for 24 h. Confocal images (single optical sections) of non-expanded tachyzoites in (**D**) versus U-ExM tachyzoites (**E**). Both were probed with anti-TgSAG1 antibody (green) and DAPI (blue), while U-ExM samples were also stained with all-protein NHS-565 dye (white). White arrows indicate the continuous host–cell environment surrounding the parasitophorous vacuole. (**F and G**) Immunofluorescence images of ME49*Δku80Δhxgprt*-infected HFFs grown under bradyzoite inducing conditions for 7 days. Confocal images (single optical sections) of non-expanded D7 cysts (**F**) versus U-ExM (**G**). Both samples were probed with anti-CST1 antibody (green) and DAPI (blue), while U-ExM samples were also stained with all-protein conjugated NHS-565 dye (white). White arrows show a gap between the fully expanded host cell and only partially expanded cyst. bz, bradyzoite; DAPI, 4′,6-diamidino-2-phenylindole; exp., expanded; tz, tachyzoite.

The standard U-ExM protocol of expansion *T. gondii* parasites from a monolayer of infected cells takes 3 days (Fig. 1A) and consists of the following steps: fixation and permeabilization of the tissue, incubation in cross-linking preventative and protein anchoring solution, gelation and polymerization, followed by denaturation at 95°C with 200-mM SDS, antibody labeling, and final expansion of the gelled sample in water (2, 52).

For the tachyzoite samples, the expansion was successful (Fig. 1B through E) with an average expansion factor of 4.3× (Fig. 1C), similar to the published values of 4.0–4.3× for *T. gondii* (10, 23, 54) *Plasmodium falciparum*, and *Cryptosporidium parvum* parasites (3).

NHS ester binds all proteins, and its fluorophore-conjugated version was used to visualize the cell architecture, which demonstrated that the host–cell environment tightly surrounds the PV in the U-ExM tachyzoites, leaving no gap as expected (Fig. 1E, white arrows). In contrast, in the U-ExM-expanded *T. gondii* cysts from D7 bradyzoite culture (Fig. 1G), a large black gap was consistently visible between the cyst, with anti-CST1 antibody used as a cyst-wall marker, and the surrounding tissue of the U-ExM-expanded host cell. Furthermore, quantification of the bradyzoite nuclei cross-section before and after expansion revealed a smaller 2.5× average expansion factor compared to 4.3× in tachyzoite samples (Fig. 1C), rendering the standard U-ExM protocol insufficient for super-resolution studies of the *Toxoplasma* cyst.

### Incubation with the mucin-selective protease StcE prior to U-ExM processing allows full expansion of the *T. gondii* cyst in fibroblasts and primary neurons

**Fig 2:**
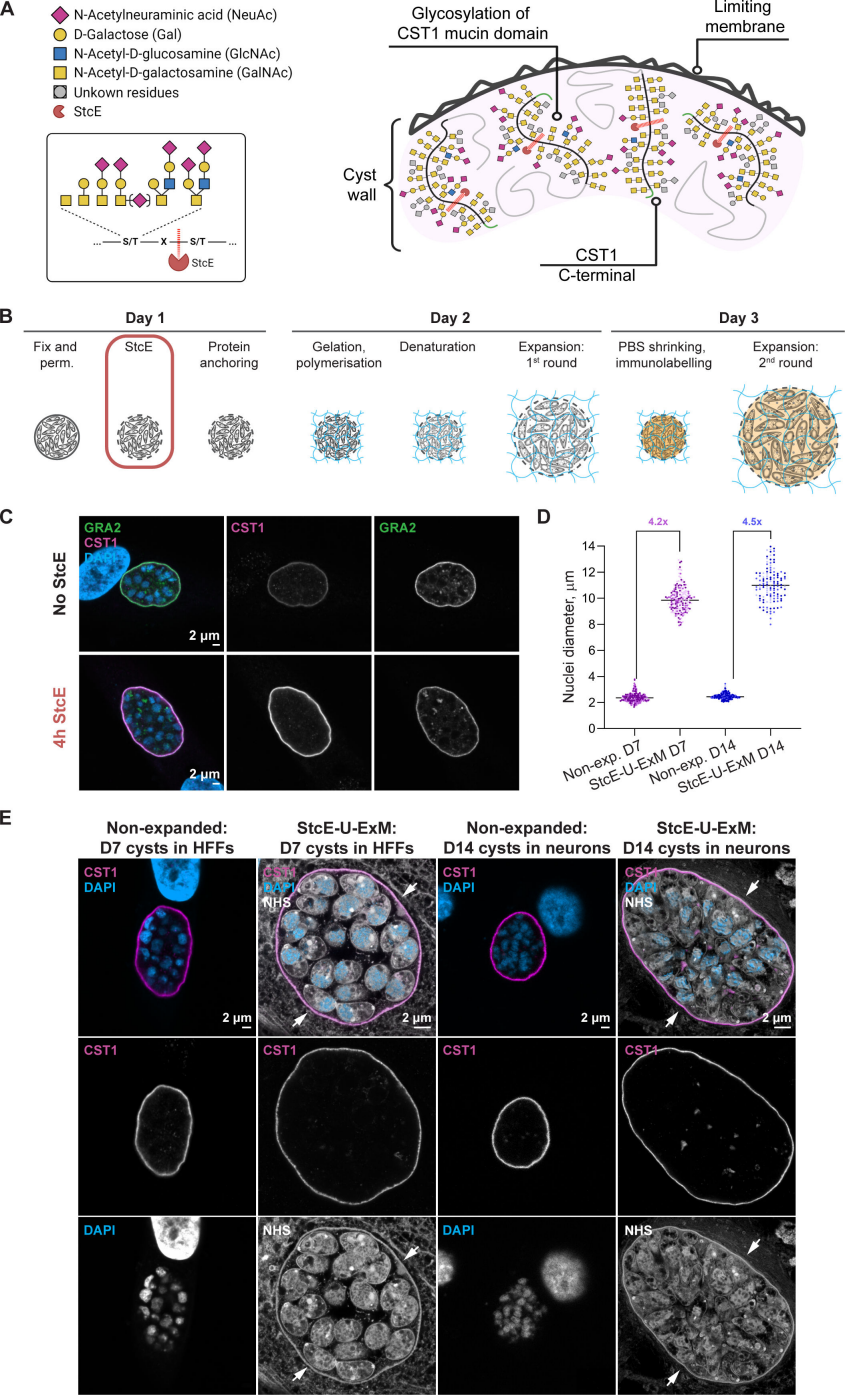
U-ExM protocol complemented with StcE digestion fully expands the cyst wall of D7 and D14 cysts. (**A**) Proposed mechanism of mucin domain cleavage by StcE mucin-selective protease. Diagram shows part of the cyst wall, depicting the limiting membrane (faces host–cell environment) and the CST1-positive layer with the ribbon-like glycoprotein structures of CST1 mucin domains. The glycoprotein structures illustrated in the cyst-wall diagram correspond to those known to be associated with CST1 [featuring GalNAc-GalNAc as a DBA-binding site (30, 36)] and those that are preferred cleavage targets of StcE. Insert depicts the StcE cleavage point on the glycopeptide chain. StcE cleaves before the second S/T within the motif S/T*–X–S/T, where the asterisk indicates modification with a glycan, and X can be any amino acid or absent (47). (**B**) Adapted U-ExM protocol workflow with added StcE enzymatic treatment (100 nM, 4 h, 37°C, no shaking) after the permeabilization step. (**C**) Representative confocal images (single optical sections) of non-expanded HFF monolayer infected with type II ME49*Δku80Δhxgprt* D7 cysts with and without StcE treatment. Samples were labeled with antibodies against GRA2 (green) and CST1 (magenta) and were stained with DAPI nuclear stain, followed by imaging under the identical acquisition settings. (Top row) No StcE treatment (control); (bottom row) samples treated with 4-h StcE at 37°C after the permeabilization step. (**D**) The expansion factor of 4.2× was determined by the comparison of the average cross-section length of D7 bradyzoite nuclei in CST1-labeled cysts stained with DAPI in non-expanded HFF samples (same non-expanded data set as in Fig. 1C) versus StcE–U-ExM HFFs and 4.5× in non-expanded neurons versus StcE–U-ExM neurons. The graph displays three independent gels or coverslips (for non-expanded samples) per condition (dots depict single measurements, and each color shade indicates measurements from the same gel) (unpaired *t*-test, *P* < 0.0001) (*n*_non-exp. bz HFFs_ = 201, *n*_non-exp. bz neurons_ = 218, *n*_exp. bz HFFs_ = 185, *n*_exp. bz neurons_ = 123). (**E**) Confocal images (single optical sections) of non-expanded D7 (in HFFs) and D14 cysts (in neurons) versus expanded using StcE–U-ExM. All samples were probed with anti-CST1 (magenta) antibody and DAPI (blue), with the additional all-protein NHS-565 stain (white) for StcE–U-ExM. White arrows indicate the continuous host–cell environment surrounding he cyst wall. Diagrams in panel **A** were created with BioRender.com. bz, bradyzoite; exp., expanded.

It is well established that the *Toxoplasma* cyst wall consists of an enrichment of glycoproteins including CST1 , SAG1-related sequence 13 (SRS13), and proteophosphoglycan TgPPG (30, 55, 56). Of these, CST1 is a major structural component of the cyst and is a SAG1-related sequence protein with a heavily glycosylated mucin domain (30). We reasoned that the dense network of glycoproteins that confer rigidity may prevent normal expansion and added a mucinase enzymatic digestion step that would disrupt glycosylated domains. StcE is a mucin-selective protease with a specific peptide- and glycan-based cleavage motif that digests the mucin domain, as shown in Fig. 2A and B (47). We first tested whether the StcE enzyme affects the proteins associated with the cyst wall in a non-expanded sample (Fig. 2C) by introducing a StcE enzymatic treatment after the permeabilization step. Cysts from the D7 bradyzoite culture labeled with antibodies against CST1 and GRA2 demonstrate the expected protein localizations after 4-h incubation with StcE at 37°C, showing that it does not destroy the cyst wall or disrupt antibody binding.

We then introduced StcE treatment after the permeabilization step in the U-ExM protocol. With this optimized protocol, we observed successful expansion of 7-day *Toxoplasma* cysts with an expansion factor of 4× (Fig. 2D), similar to that of tachyzoites. Crucially, no gap is observed between the StcE–U-ExM cyst and the host–cell environment (Fig. 2E, white arrows).

**Fig 3:**
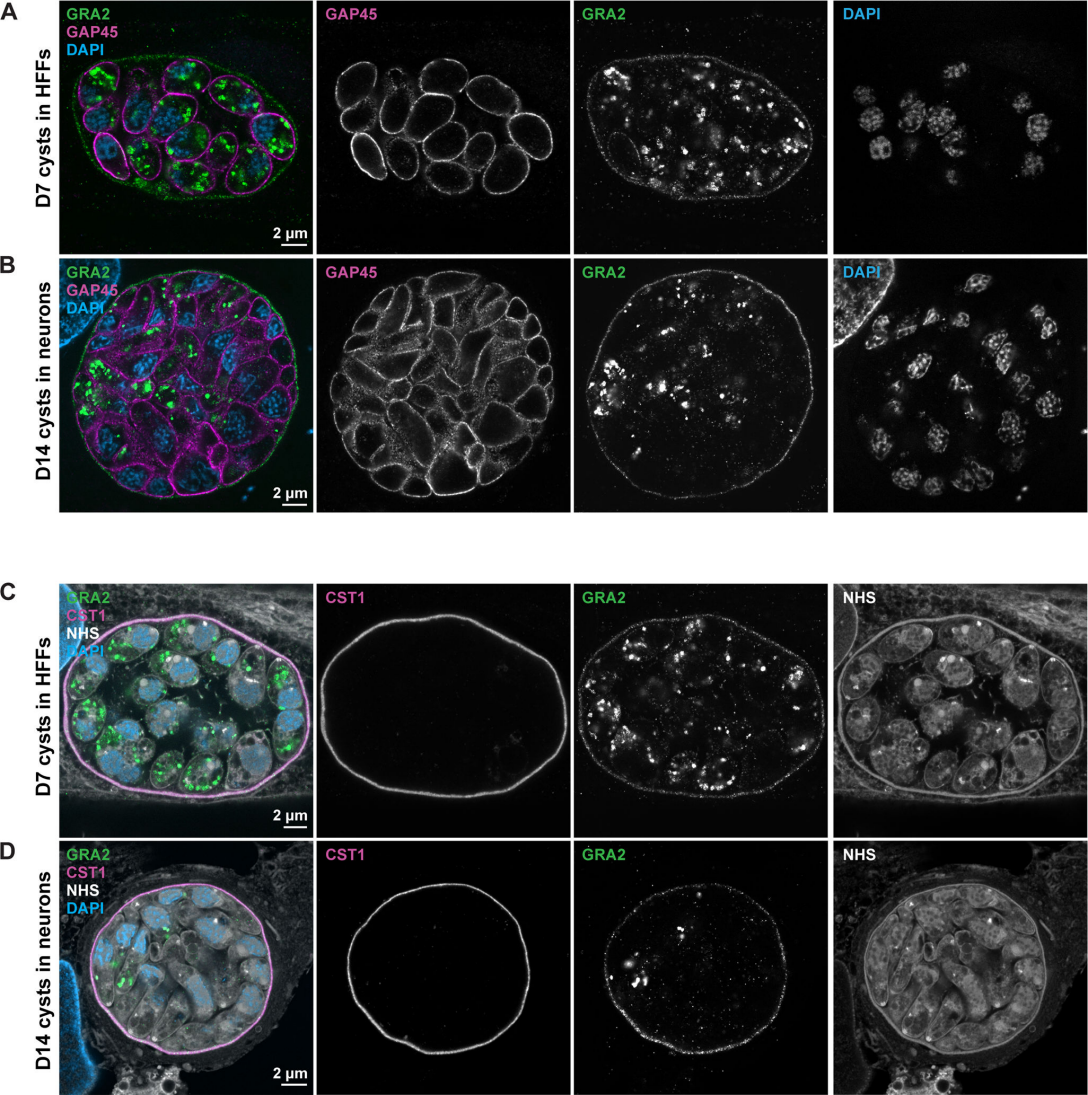
StcE–U-ExM protocol preserves protein signals in *T. gondii* cysts *in vitro* (D7 fibroblasts and D14 neurons). (**A and B**) Confocal image (single optical section) of the StcE–U-ExM HFF monolayer with type II ME49*Δku80Δhxgprt* D7 cyst (**A**) and primary neuronal culture (derived from P0 rat hippocampus) with type II 76K D14 cyst (**B**) at ×100 magnification. Samples were labeled with antibodies against GRA2 (green), GAP45 (magenta), and biotinylated DBA (not shown) and were stained with DAPI nuclear stain (blue). Each row consists of a merged image on the left, followed by separate channels in gray. (**C and D**) Confocal image (single optical section) of the StcE–U-ExM HFF monolayer with type II ME49*Δku80Δhxgprt* D7 cyst (**C**) and primary neuronal culture (derived from P0 rat hippocampus) with type II 76K D14 cyst (**D**) at ×100 magnification. Samples were labeled with antibodies against GRA2 (green) and CST1 (magenta) and were stained with NHS-565 (white) and DAPI (blue).

It was recently shown that D14 *in vitro* cysts were able to infect a mouse following oral gavage, while infection with D7 cysts was unsuccessful (33). This may be that the cyst wall continues to mature and becomes more robust over time or that the parasites differentiate further. In order to verify the StcE-enhanced U-ExM protocol on infectious cysts, we tested 14-day-old cysts generated in primary neuronal cultures from postnatal rat hippocampus alongside the D7 cysts from the HFF monolayer. Reassuringly, a similar expansion factor was observed (Fig. 2D), with no gap between the packed cyst structure and the surrounding cell (Fig. 2E). To further assess the method, StcE–U-ExM *in vitro* cysts were probed with antibodies against the inner membrane complex, GAP45, and the secreted protein GRA2 along with anti-CST1 for the cyst wall (Fig. 3A through D). All antibodies showed their expected localization patterns: GAP45 outlined the parasite shape; GRA2 was split between intracellular punctate staining, presumably within dense granules, and the periphery of the cyst; and CST1 localized around the cyst periphery. Furthermore, StcE did not cause any observable alterations in the subcellular ultrastructure of bradyzoites when assessed using NHS-conjugated ester in D7 cysts (Fig. S2; Movie S1).

### Modified U-ExM-StcE protocol reveals that GRA2 is only partially localized to the CST1-positive layer in the cyst wall

**Fig 4:**
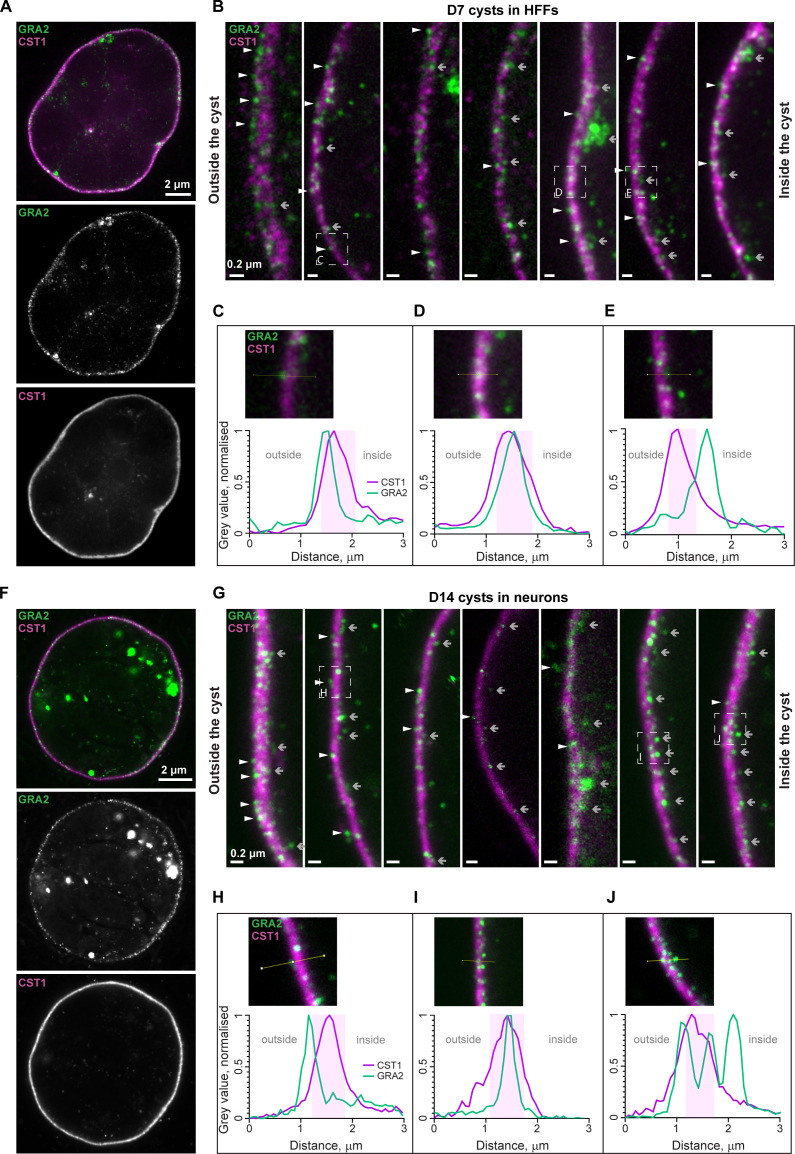
U-ExM of *in vitro* cysts show GRA2 spanning the CST1-positive layer in HFF D7 and neuronal D14 *T. gondii* culture. (**A**) Confocal image of the StcE–U-ExM HFF monolayer with type II ME49*Δku80Δhxgprt* D7 cyst at ×100 magnification (single optical section of the widest cross-section of the cyst). Sample was labeled with antibodies against GRA2 (green) and CST1 (magenta). (**B**) Zoomed cyst-wall regions selected from the three D7 cysts (all scale bars 0.2 µm). Arrowheads (gray) and arrows (white) highlight examples of GRA2 puncta inside and outside the CST1 layer, respectively. Boxes indicate displayed areas in panels **C–E**. (**C–E**) Fluorescence intensity profiles showing different locations of GRA2 relative to CST1 in a single StcE–U-ExM D7 cyst grown in the HFF monolayer. Normalized intensity levels (gray values) obtained from the raw images were plotted against the length of the sampled area (yellow line, image insert). The shaded area indicates the relative boundaries of the CST1 layer, defined using 50% of the signal intensity around the mean, assuming a normal distribution. (**C**) GRA2 localizes outside the CST1 layer, on the exterior side of the cyst wall, facing host–cell environment. (**D**) GRA2 localizes within the CST1 layer. (**E**) GRA2 localizes outside the CST1 layer, on the interior side of the cyst wall facing the cyst matrix. (**F**) Confocal image of the StcE–U-ExM primary neuronal culture (derived from P0 rat hippocampus) with type II 76K D14 cyst at ×100 magnification (single optical section of the widest cross-section of the cyst), presented as in panel** A**. (**G**) Zoomed cyst-wall regions selected from the three D14 cysts (all scale bars 0.2 µm). Arrows, arrowheads, and boxes as in panel** B**. See also Movie S2. (**H–J**) Fluorescence intensity profiles showing different locations of GRA2 relative to CST1 in a single StcE–U-ExM type II 76K D14 cyst grown in a primary neuronal culture. Normalized intensity levels (gray values) obtained from the raw images were plotted against the length of the sampled area (yellow line, image insert). (**H**) GRA2 localizes outside the CST1 layer, on the exterior side of the cyst wall facing host cell environment. (**I**) GRA2 localizes within the CST1 layer. (**J**) Three GRA2 punctae localize across the CST1 layer: one on the exterior side of the cyst wall, one in the middle of CST1-positive layer, and one outside, facing the cyst matrix. Shaded areas indicate the relative boundaries of the CST1 layer, as in panels** C–E**.

While many proteins secreted from the dense granules are known to localize to the cyst wall (46, 57, 58), current microscopy techniques have not allowed a more detailed visualization of cyst wall substructures. We therefore used the StcE–U-ExM protocol to clarify the location of GRA2 relative to CST1 protein in the *T. gondii* cyst wall in HFFs (D7) and primary rat neuronal cultures (D14). While previous data have shown a smooth overlapping signal for CST1 and GRA2 in the *in vitro* cyst wall (38), we observe a more punctate distribution of GRA2 in the cyst wall compared to the smooth signal of CST1 with StcE-enhanced U-ExM (Fig. 4A).

To confirm that the observed GRA2 puncta are not an imaging artifact or background signal as occasionally observed with ExM protocols, we compared an alternative mouse primary antibody and a secondary antibody-only control. Matrix antigen 1 (MAG1) is a *Toxoplasma* secreted protein that localizes throughout the matrix of the cyst in addition to the cyst wall (59). Anti-MAG1 mouse primary antibody paired with AlexaFluor 488 anti-mouse (as used in Fig. 2 to 4 with anti-GRA2 primary mouse antibody) showed a disperse protein distribution throughout the cyst, as expected, lacking any puncta (Fig. S3A). Reassuringly, no puncta were visible in the secondary antibody-only control using the same image acquisition settings (Fig. S3B), further confirming that the observed puncta are GRA2 specific.

The GRA2 granules in the StcE–U-ExM cyst are observed on both the outer and inner sides of the CST1 layer, with enrichments visible on the inner side of the wall (Fig. 4B through E). In viewing the z-stack of images through the wall, these GRA2 structures appear to span through the CST1-positive layer (Movie S2). A similar pattern was observed in D14 cysts from infected primary neuronal cultures (Fig. 4F through J).

## DISCUSSION

Despite the importance of chronic *Toxoplasma* cysts in transmission and their reactivation in human disease, the detailed structure of the cyst is yet to be clarified. While EM has provided key insights into the wall morphology, the resolution of immuno-EM has limited the ability to precisely localize many cyst-wall proteins. A relatively new modification of the sample expansion technique, U-ExM, is an easy, cost-effective alternative to the established super-resolution approaches, allowing linking protein location to the sample morphology on the same sample.

When attempting to use U-ExM for cyst-wall protein localization, we discovered that the standard U-ExM protocol fails to expand the cyst wall up to the expected 4.0–4.3× expansion factor. The addition of the enzymatic step targeting glycosylated mucin domains of CST1 and, potentially, SRS13 proteins using StcE mucinase solves the ineffective expansion, allowing protein localization studies in fully expanded cysts. We confirmed our results in day 7 and day 14 cysts grown in HFF monolayers and primary rat neuronal cell culture, respectively.

We then used the technique to clarify the location of the GRA2 protein relative to the CST1-positive layer in the cyst wall. Previously published studies based on standard confocal images show full co-localization of GRA2 and CST1, both appearing as a smooth signal across the cyst wall (38). Using a modified StcE–U-ExM protocol, we show that GRA2 is distributed as puncta across the still smooth CST1-positive layer, which was consistent in both in D7 *Toxoplasma* cysts in fibroblasts and D14 cysts in neurons *in vitro*. Moreover, fully expanded StcE–U-ExM cysts show GRA2 spanning the CST1-positive layer, providing previously unknown details about the GRA2 location in the *Toxoplasma* cyst. Enrichments of GRA2 signal were observed underneath the CST1 layer, with putative spirals going through the CST1 layer. In the future, it will be interesting to investigate if these represent clusters of the tubular network of the IVN and colocalize with other GRAs.

Early work on GRA2 localization using immunoelectron microscopy with labeled golden particles showed GRA2 secretion from the tachyzoites into the PV lumen and its association with the IVN, although the GRA2 localization does not follow any specific pattern (60). It is well known that GRA2 is essential for the correct organization of the IVN in tachyzoite PVs (43) and impacts the cyst wall and matrix during the chronic stage (38). This, as well as its relocation to the cyst wall with the subsequent co-localization with the major structural protein CST1, suggests that GRA2 retains its role in the organization of the parasitic structures after stage conversion. However, its precise function is still unclear.

Although some GRA2 remains in the cyst matrix as puncta, a significant portion of it relocalizes to the cyst wall (38). Regular (non-expansion) immunofluorescence assays depict cyst wall-associated GRA2 as a smooth continuous signal. We show that, after expansion, GRA2 was distributed in puncta in the cyst wall.

Despite the growing number of proteins localized to the cyst wall over the past few years (57, 58, 61), we still do not know their functional relevance in the development and maturation of the cyst. By revealing more precise protein localizations, StcE–U-ExM can play a key role in investigating their role by increasing our knowledge of substructures within the wall. Indeed, with the growth of *Toxoplasma* cysts over time and their subsequent rupture to release parasites, it is clear that the cyst wall is subject to remodeling.

In conclusion, the StcE–U-ExM protocol is a powerful addition to the microscopy toolbox aimed at the investigation of *in vitro Toxoplasma* cysts with nanoscale resolution, joining other recent efforts to adapt expansion protocols for non-mammalian samples via tailored enzymatic disruption (62). In our case, the simplicity of a single-enzyme treatment with a pan-mucinase (49) to break down the mucin-rich glycocalyx of the *Toxoplasma* cyst wall underscores the importance of biochemical characterization of structural biomolecules for successful expansion microscopy.

## Data Availability

All data is freely available upon request.

## SUPPLEMENTAL MATERIAL

Supplemental Material msphere.00322-24-s0001.docx Fig. S1-S3; supplemental movie legends.

Movie S1 msphere.00322-24-s0002.mp4 Intracellular bradyzoite structures in the StcE-U-ExM D7 cyst in HFF monolayer.

Movie S2 msphere.00322-24-s0003.mp4 The GRA2 granules in the StcE-U-ExM cyst span through the CST1-positive layer.

ASM does not own the copyrights to Supplemental Material that may be linked to, or accessed through, an article. The authors have granted ASM a non-exclusive, world-wide license to publish the Supplemental Material files. Please contact the corresponding author directly for reuse.
