# Supplementary material for: Enzymatically enhanced ultrastructure expansion microscopy unlocks expansion of in vitro Toxoplasma gondii cysts
Source: mSphere. 2024 Aug 27;9(9):e00322-24. doi: 10.1128/msphere.00322-24 (PMC11423595; doi:10.1128/msphere.00322-24)
Supplement: Supplemental Material — Fig. S1-S3; supplemental movie legends. [file msphere.00322-24-s0001.docx]

# Supplementary material


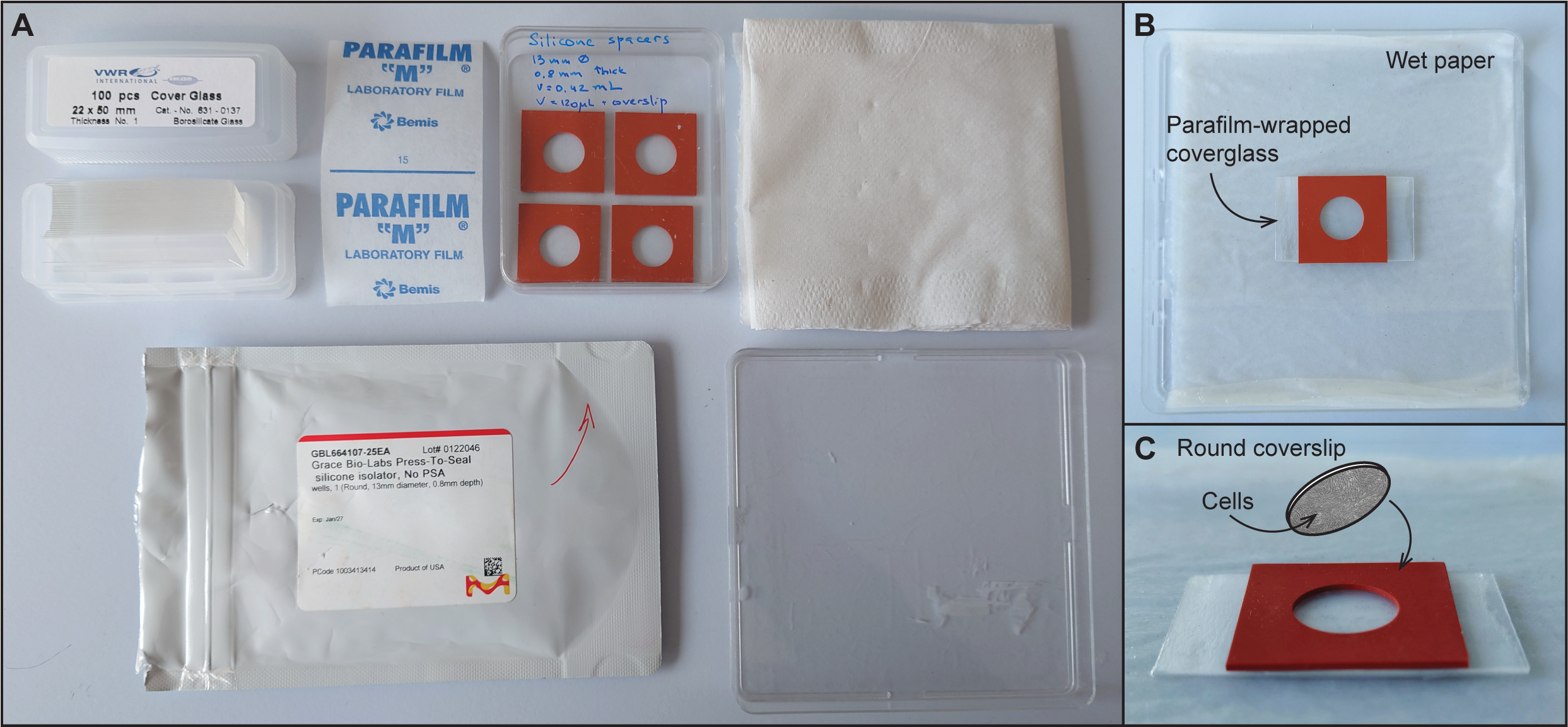


**Figure S1. Gelation chamber set-up**

**(A)** Gelation chamber components. Coverglass (any), parafilm, round 13mm diameter silicone spacers (could be any but we used Grace Bio-Labs GBL664107-25EA press-to-seal), plastic dish and paper to line it. **(B)** Three coverglasses were stacked together and wrapped in a parafilm. The silicone spacer is pressed on top. They were then placed on a straightened wetted paper in the plastic dish, moved to –20°C freezer for 5 min, and then kept on ice. **(C)** The silicone well is then filled with 120 µL of fresh gelation solution and the round sample coverslip is placed over it, cell side facing down.


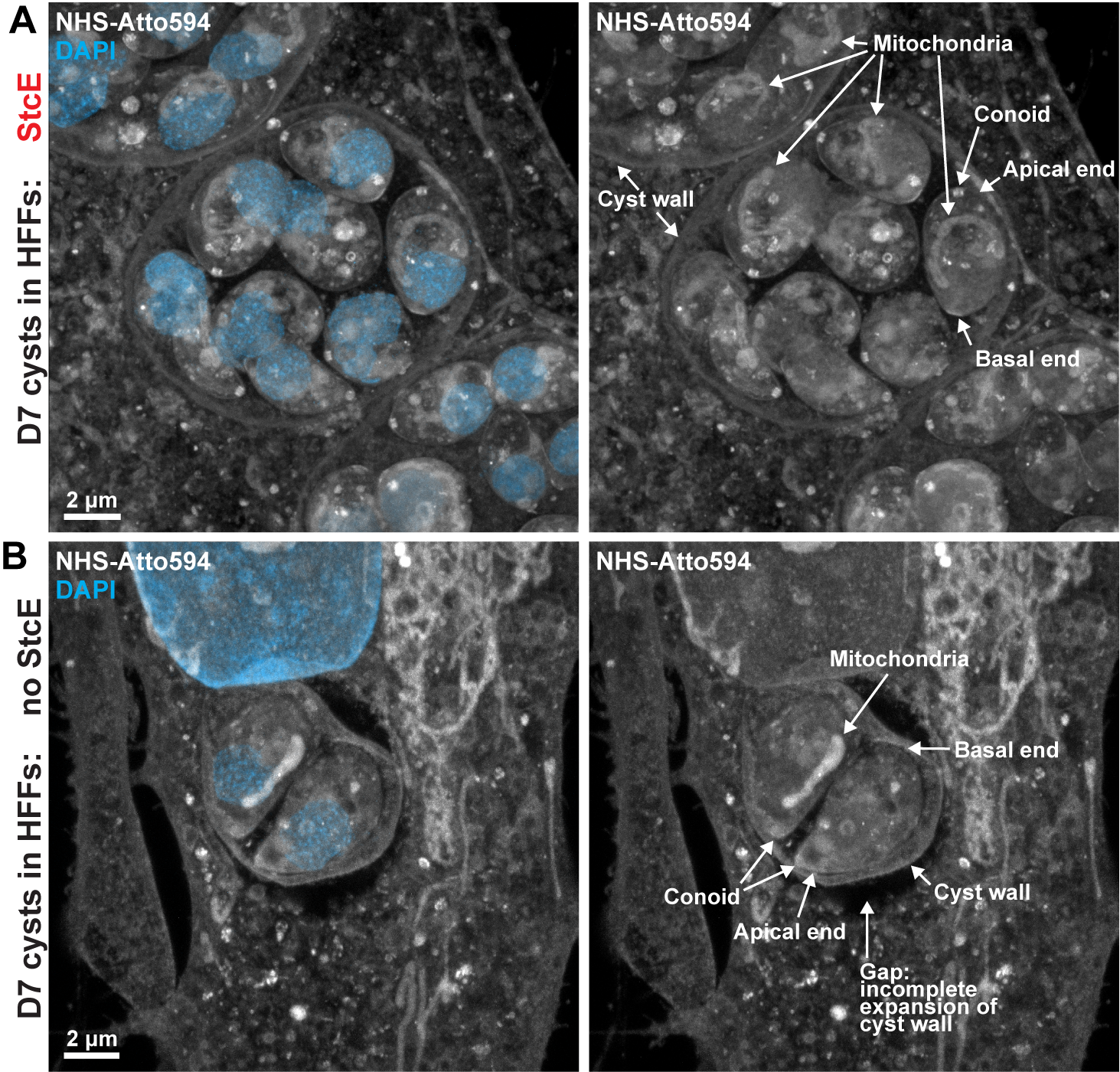


**Figure S2. StcE treatment preserves parasite ultrastructure in StcE-U-ExM samples**

**(A)** Confocal image of the StcE-U-ExM HFF monolayer with Type II ME49Δku80Δhxgprt D7 cyst at 100x magnification (single optical section of the widest cross-section of the cyst). Sample was labelled with NHS-Atto594 (white) and DAPI (blue). **(B)** Confocal image of the U-ExM HFF monolayer with Type II ME49Δku80Δhxgprt D7 cyst at 100x magnification (single optical section of the widest cross-section of the cyst). Sample was labelled with NHS-Atto594 (white) and DAPI (blue). (A-B) Parasite orientation and its organelles (highlighted using white arrows) were identified by similarity to the electron microscope images of bradyzoites (1), standard fluorescence images of bradyzoites (2), and super-resolution microscopy images of the mitochondrial lasso morphology in tachyzoites (3).


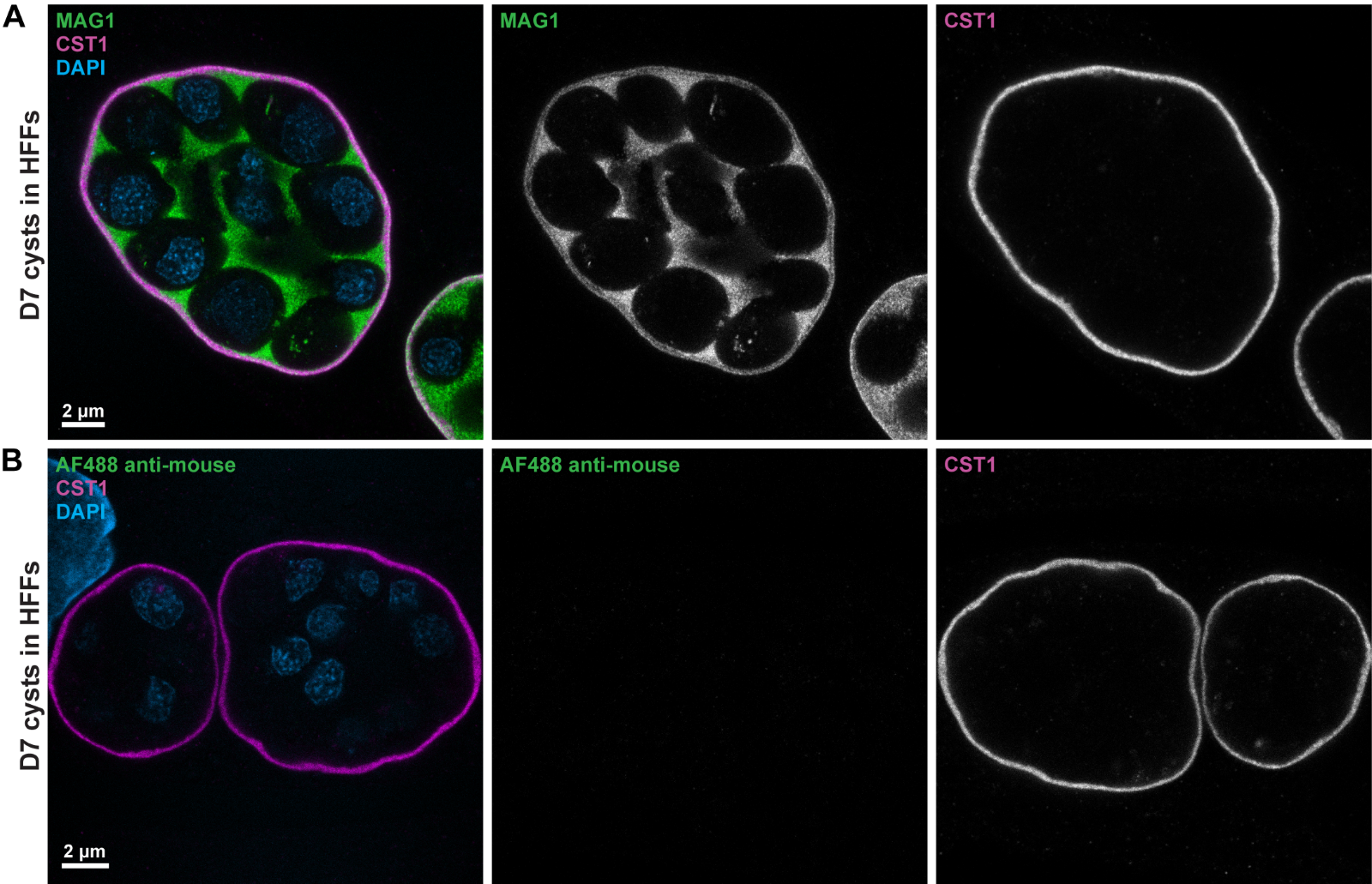


**Figure S3. Secondary antibody validation**

**(A)** Confocal image of the StcE-U-ExM HFF monolayer with Type II ME49Δku80Δhxgprt D7 cyst at 100x magnification (single optical section of the widest cross-section of the cyst). Sample was labelled with unconjugated primary antibodies against MAG1 (mouse, green) and CST1 (rabbit, magenta), and then with DAPI and two secondary antibodies, Alexa Fluor 488 anti-mouse (used in Fig.2-4 for anti-GRA2 unconjugated primary mouse antibody) and Atto647 anti-rabbit. **(B)** Confocal image of the StcE-U-ExM HFF monolayer with Type II ME49Δku80Δhxgprt D7 cyst at 100x magnification (single optical section of the widest cross-section of the cyst). Sample was first incubated with unconjugated primary antibody against CST1 (rabbit, magenta), and then with two secondary antibodies, Alexa Fluor 488 anti-mouse (used in Fig. 2-4 for anti-GRA2 unconjugated primary mouse antibody) and Atto647 anti-rabbit.

**Movie S1**

**Intracellular bradyzoite structures in the StcE-U-ExM D7 cyst in HFF monolayer**

ME49Δku80Δhxgprt bradyzoite cysts grown in HFFs for 7 days, stained with NHS-Atto594 all-protein stain and DAPI, and expanded using StcE-U-ExM protocol. The video shows three cysts in the human foreskin fibroblast, with the focus on the central one.

**Movie S2**

**The GRA2 granules in the StcE-U-ExM cyst span through the CST1-positive layer.**

ME49Δku80Δhxgprt bradyzoite cysts grown in HFFs for 7 days, probed with antibodies against GRA2 (green) and CST1 (magenta), and expanded using StcE-U-ExM protocol as in Figure 4. The video shows the z axis of a zoomed-in part of the three expanded cyst walls facing the host cell environment (left hand side). The arrows indicate the direction of movement along z axis. The “up” arrow indicate the movement away from the glass coverglass towards the top of the cyst, and the “down” arrow vice versa. The video demonstrates GRA2 puncta “spanning” in or around the CST1-positive layer of the cyst wall.

**References**

1. Dubey JP, Lindsay DS, Speer C. Structures of Toxoplasma gondii tachyzoites, bradyzoites, and sporozoites and biology and development of tissue cysts. Clinical microbiology reviews. 1998 Apr 1;11(2):267-99.

2. Place BC, Troublefield CA, Murphy RD, Sinai AP, Patwardhan AR. Machine learning based classification of mitochondrial morphologies from fluorescence microscopy images of Toxoplasma gondii cysts. PLoS One. 2023 Feb 2;18(2):e0280746.

3. Ovciarikova J, Lemgruber L, Stilger KL, Sullivan WJ, Sheiner L. Mitochondrial behaviour throughout the lytic cycle of Toxoplasma gondii. Scientific reports. 2017 Feb 16;7(1):42746.
